# Initial experience of complete laparoscopic radical nephroureterectomy combined with transvesical laparoscopic excision of distal ureter in patients with upper urinary tract cancer

**DOI:** 10.1186/s12957-020-01872-1

**Published:** 2020-05-25

**Authors:** Makito Miyake, Nobutaka Nishimura, Katsuya Aoki, Chihiro Ohmori, Takuto Shimizu, Takuya Owari, Shunta Hori, Yosuke Morizawa, Daisuke Gotoh, Yasushi Nakai, Satoshi Anai, Kazumasa Torimoto, Nobumichi Tanaka, Kiyohide Fujimoto

**Affiliations:** Department of Urology, Nara Medical University, 840 Shijo-cho, Kashihara, Nara, 634-8522 Japan

**Keywords:** Upper urinary tract urothelial cancer, Transvesical laparoscopy, Complete laparoscopy, Pneumovesicum, Numeric pain rating scale

## Abstract

**Background:**

Selecting the treatment procedure for cancer patients is a challenging task. We report our initial experience of complete laparoscopic radical nephroureterectomy (RNU) for patients with upper urinary tract urothelial cancer (UTUC).

**Methods:**

A total of four patients with UTUC underwent complete laparoscopic RNU combined with transvesical laparoscopic excision of the distal ureter using three 5-mm ports. Transvaginal specimen extraction was applied in female patients to reduce incisional pain and improve cosmesis. Peri-operative complications were evaluated using the Clavien-Dindo classification system. Postoperative pain was evaluated during hospitalization using a numeric pain rating scale (scales of 1 to 10). Patients who underwent retroperitoneal laparoscopic surgery combined with open excision of the distal ureter during the same period were included as a control group (conventional RNU, consisting of laparoscopic nephrectomy combined with open bladder cuff excision) for pain scale evaluation.

**Results:**

The novel surgery was successfully completed for all four patients (two males and two females). The mean pneumoperitoneum time for retroperitoneoscopic nephroureterectomy and specimen extraction was 174 min, while the mean pneumovesicum time for the ureteral orifice excision was 88 min. One male patient had bladder leakage at the suture site of the bladder wall, which lasted for 2 weeks. No patient experienced recurrent disease during the follow-up period (median, 10 months). Mild to moderate pain lasted for 5 or 6 days after RNU. A couple of days after surgery, the numeric pain rating scale of complete laparoscopic RNU and conventional RNU group reached its peak level at 3.0 ± 1.8 and 5.3 ± 2.8, respectively. There was no statistical difference in the degree of postoperative pain (*P* = 0.31).

**Conclusions:**

We described our initial experience and outcome of complete laparoscopic RNU for UTUC. Further experience and research are required to determine whether this advanced laparoscopic technique yields better outcomes and has true clinical value.

## Introduction

Selecting the best treatment procedure for cancer patients in the clinical practice is a challenging task. Radical nephroureterectomy (RNU) with complete removal of the distal ureter, including ureteral orifice (bladder cuff excision), is the standard surgical treatment for upper urinary tract urothelial cancer (UTUC) [1, 2]. Although open RNU was commonly performed before 1990, minimally invasive laparoscopic surgery has rapidly evolved since 1991, with the first reports of laparoscopic RNU being reported by Clayman et al. [3]. Despite the lack of well-designed prospective randomized control trials, meta-analysis-based comparisons between laparoscopic RNU and its open counterpart demonstrated that both offer equivalent outcomes in terms of oncological efficacy, peri-operative safety, and mortality [4, 5].

To date, laparoscopic RNU has been broadly accepted by urologists mainly due to reduced postoperative pain and improved cosmetic results. Many urologists select a combination of excision of the kidney and upper ureter by retroperitoneoscopic approach and bladder cuff excision by lower abdominal incision-open surgery. This combination is not a complete laparoscopic surgery and thus is not a minimally invasive method. The best method for complete laparoscopic RNU for patients with UTUC has not yet been established. The approaches for bladder cuff excision are still controversial and include intravesical, extravesical, and transurethral incisions [6]. The extravesical technique carries a potential risk of residual tumor at the distal intramural part of the ureter, while the intravesical technique carries the risk of tumor cell dissemination to the outside of the bladder.

Here, we report the initial experience of complete laparoscopic nephroureterectomy with transvesical laparoscopic excision of the distal ureter using three ports in patients with UTUC. The transvesical laparoscopic approach is an alternative to open surgery in children with vesicoureteral reflux and bladder diverticulum [7, 8]. Transvaginal specimen extraction was applied in the case of female patients to reduce incisional pain and improve cosmesis.

## Patients and methods

### Clinical data

This research was approved by the ethics committee of the Nara Medical University, and all participants provided informed consent (reference ID: 1256 and 1719). The date of ethical approval was May 30, 2017, which is prior to the first surgery performed. We prospectively selected the patients and gave full information regarding the novel surgical method. All patients underwent dynamic computed tomography (CT) or CT urography before surgery to determine tumor location and size. The main exclusion criteria were (1) tumor of the distal ureter, (2) advanced tumor detected by CT (suspected T3/T4 disease or node-positive disease), (3) contraindications to laparoscopic surgery, or (4) concurrent bladder tumor.

We conducted a review of four patients with UTUC who underwent complete laparoscopic nephroureterectomy with transvesical laparoscopic bladder cuff excision between January 2018 and December 2019. All surgeries were performed by a single laparoscopically experienced surgeon (M. Miyake). Peri-operative complications were objectively evaluated using the Clavien-Dindo classification system [9]. This system includes seven grades (I, II, IIIa, IIIb, IVa, IVb, and V). The degree of postoperative pain was evaluated every day during hospitalization and on the first outpatient visit day using a numeric pain rating scale (NPRS), where 0 indicates no pain and 10 indicates the worst imaginable pain [10]. Four patients who underwent conventional retroperitoneal laparoscopic surgery combined with an open bladder cuff using a lower abdominal midline incision, by the same surgeon (M. Miyake), during the same period, were included as a control group for pain scale evaluation.

### Surgical procedure for complete laparoscopic RNU

**Fig. 1:**
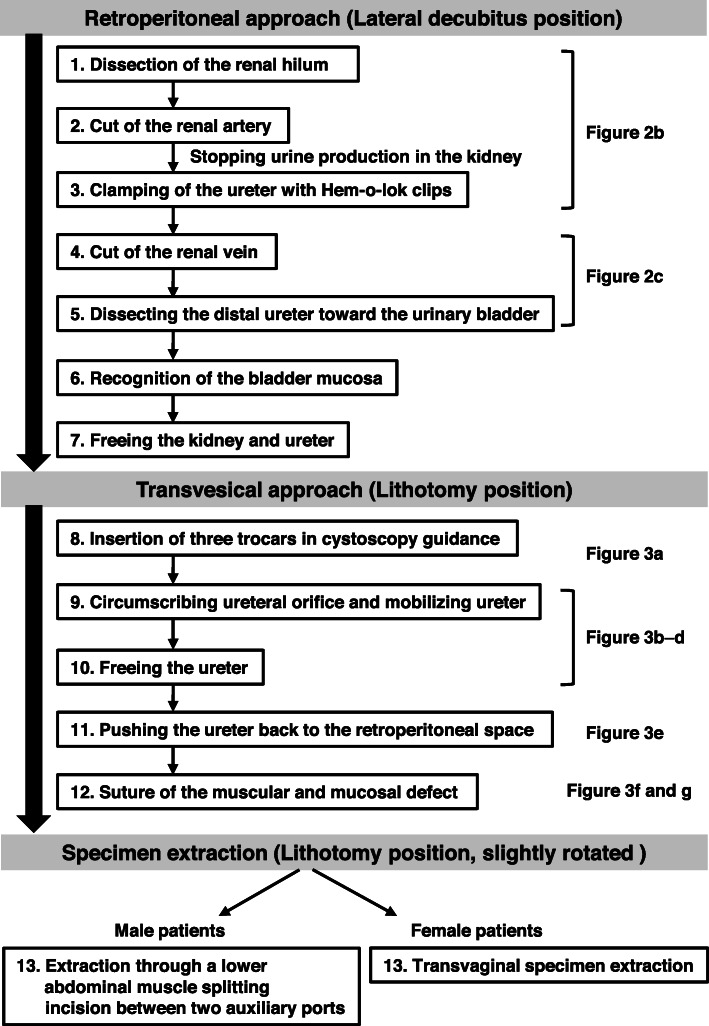
Diagram of the surgical procedure

**Fig. 2:**
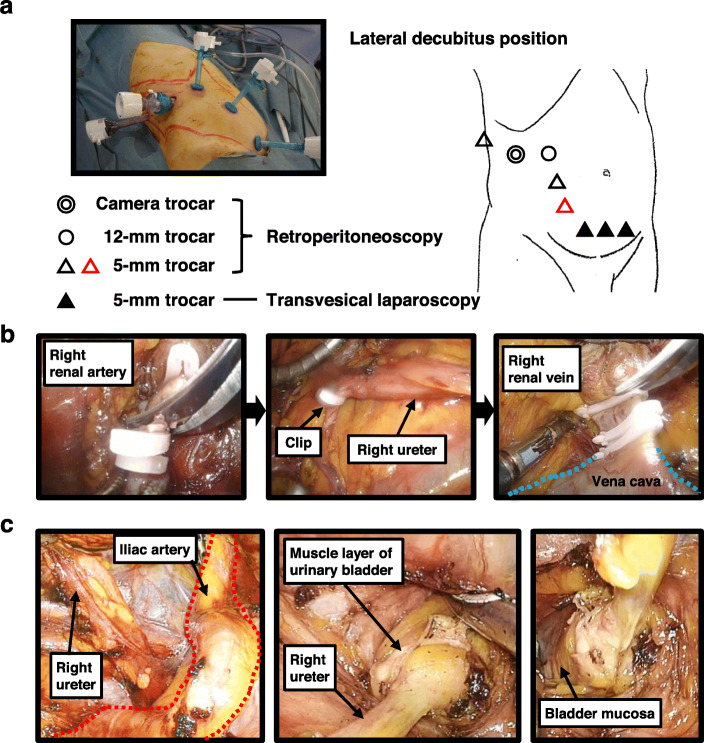
Representative image (case 4 in Table 1) of a patient undergoing complete laparoscopic nephroureterectomy. **a** Trocar positions for nephroureterectomy of a right-side UTUC. The lower-level auxiliary port (red open triangle) is added to provide a wide surgical view of the pelvic area. Suprapubic ports (black triangles) are used for transvesical laparoscopic bladder cuff excision. **b** The cut of the renal artery, clamping of the ureter, and cut of the renal vein were performed with Hem-o-lok® clips. **c** Pulling up the proximal ureter to assist in dissecting the distal ureter toward the urinary bladder. When the junction of the ureter and bladder is exposed, the muscle layer is cut off, followed by recognition of the bladder mucosa


An operation video demonstrating the surgical procedures is given in Supplementary Video S1. A diagram of each step of the surgical procedure is depicted in Fig. 1. Under general anesthesia, each patient was placed in a lateral decubitus position with the cancerous side up for a retroperitoneoscopic nephrectomy. The retroperitoneal cavity was dilated with a retroperitoneal balloon and maintained with 8 mmHg CO_2_ of insufflation pressure. We carried out conventional retroperitoneal approach with four ports as shown in Fig. 2a: a flexible endoscope (camera) trocar, 12-mm trocar, 5-mm trocar, and auxiliary 5-mm trocar. The procedure includes the standard nephrectomy using laparoscopic monopolar scissors (e.g., AESCULAP® laparoscopic instruments), LigaSure™ Maryland jaw sealer (Covidien Japan, Tokyo, Japan), and Hem-o-lok® clips [11]. The renal artery was secured with a size L clip, followed by clamping of the ureter at the distal area with a size L clip or ML clip after stopping urine production in the kidney (Fig. 2b). Then, the renal vein was secured with a size XL clip, and the kidney was completely freed. The adrenal gland was retained in all cases.

**Additional file 1: Supplementary Video S1:** Operation video demonstrating the surgical procedures.


A 5-mm trocar was added to the ipsilateral pelvic area to facilitate a wide operation space in the phase of dissection of the distal ureter (Fig. 2a, red triangle). The ureter was dissected under the common iliac artery to the bladder. The distal ureter was dissected maximally, and the urinary bladder remained unclosed during this phase (Fig. 2c). During the retroperitoneoscopic procedure, the ureter was not cut to enable complete en bloc removal of the kidney and ureter.

**Fig. 3:**
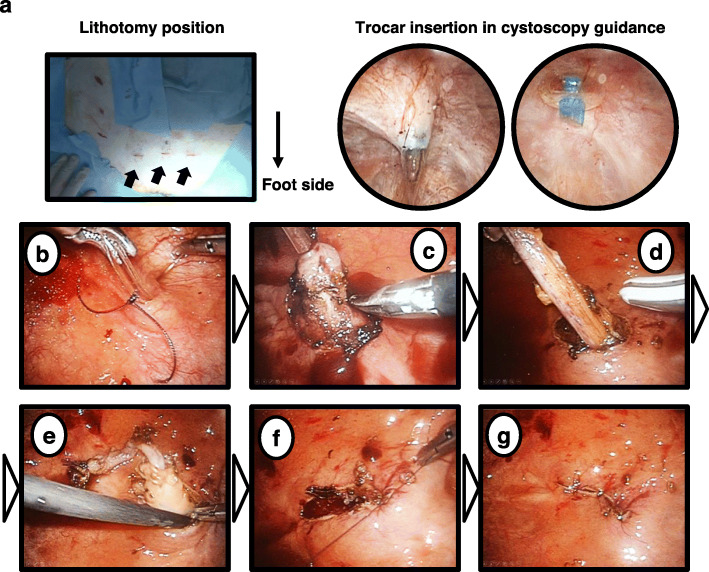
Procedure of transvesical laparoscopic bladder cuff excision. **a** Postoperative wound for the suprapubic three ports is shown (black arrows). The bladder was distended with 400–500 mL of saline. A total of three 5-mm trocars were placed at the bladder dome and on both sides of the lateral wall of the distended bladder under cystoscopy guidance. A 3-0 monofilament traction suture is passed percutaneously through the bladder walls to prevent the bladder wall from falling away from the abdominal wall. **b** A 4-cm-long segment of an 8Fr pediatric feeding tube is inserted into the ipsilateral ureter to facilitate ureteral mobilization and dissection and secured by a 5-zero monofilament suture. **c** Circumscribing ureteral orifice and mobilizing ureter using fine 3-mm endoscopic scissors. **d** Traction on the ureteric catheter and cut of fibrovascular tissue surrounding the ureter to free it. **e** The ureter is pushed back to the retroperitoneal space. **f** The muscular defect and mucosal defect in the ureteral hiatus are sutured intravesically using 5-zero absorbable monofilament sutures, usually with an extracorporeal knot-tying technique. **g** Complete suturing of the bladder wall defect

Next, the patient was changed to the lithotomy position for transvesical laparoscopic bladder cuff excision. The operation was restarted with cystoscopy and performed according to the procedure reported by Yeung et al. [12]. The surgeon stands on the patient’s left side. Under cystoscopic guidance, three 5-mm trocars (Kii Advanced Fixation Sleeve; Applied Medical, Rancho Santa Margarita, CA, USA) were placed from the suprapubic region into the bladder (Fig. 3a). Under 8 mmHg CO_2_ of pneumovesicum pressure, the ureterovesical junction and Waldeyer’s sheath were excised with a 3-mm laparoscopic monopolar scissors until the paravesical adipose tissue was visible (Fig. 3b–d). After completely mobilizing the ureter, the ureter was pushed back to the retroperitoneal space (Fig. 3e). Then, the muscular defect and mucosal defect in the ureteral hiatus were sutured intravesically (Fig. 3f, g). Intravesical trocars were removed under endoscopic vision without suturing the bladder wall. Each port site entry wound was closed with a 4-0 PDSII monocryl suture (Ethicon, NJ, USA).

**Fig. 4:**
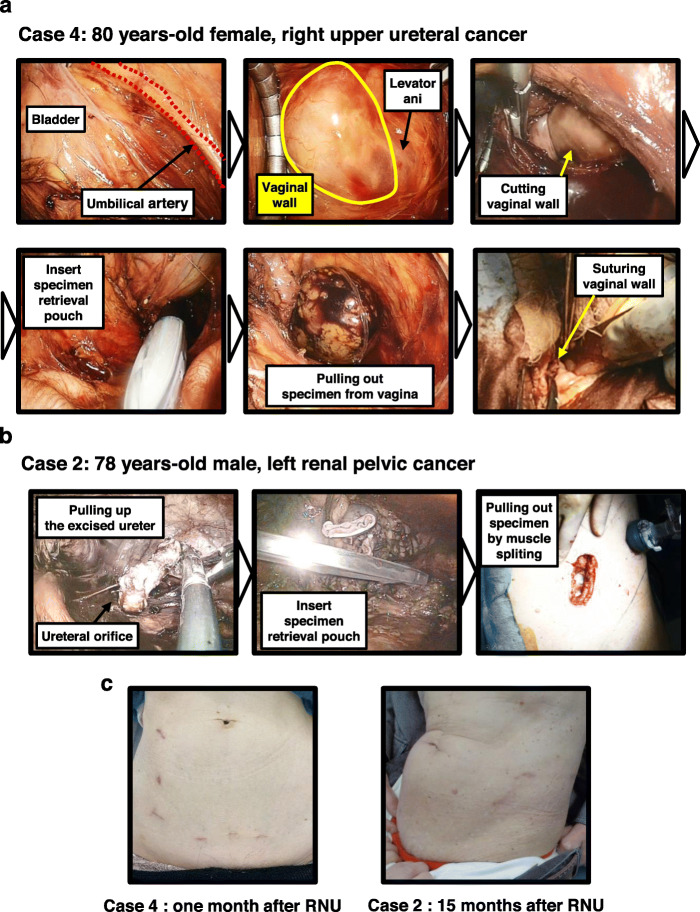
Procedure of en bloc tissue extraction: two representative cases. **a** Transvaginal specimen extraction was applied in an 80-year-old patient with right upper ureter cancer. The specimen was packed in EndoCatch™ II specimen retrieval pouch (Medtoronic, Minneapolis, MN, USA) through the vaginal side wall. After pulling out the specimen, the vaginal wall was closed with absorbable surgical suture. **b** In male patients, the specimen was extracted in a retrieval pouch through a lower abdominal muscle splitting incision between two auxiliary ports. **c** Photographs of postoperative wounds in two representative cases

In female patients, the specimen was extracted transvaginally in a bag (Fig. 4a). In male patients, the specimen was extracted in a bag through a lower abdominal muscle splitting incision between the two auxiliary ports. A pelvic drain tube was placed through the pelvic auxiliary port.

### Control group undergoing conventional RNU

Conventional surgery consists of laparoscopic nephrectomy with open bladder cuff excision. In the retroperitoneoscopic phase, the ureter was freed to the bifurcation level of iliac vessels along the level of the lower pole of the kidney. Next, the patient was placed in the supine position for open bladder cuff excision. An 8–10-cm midline incision was made on the lower abdomen. After extracting the kidney from the body, pulling the ureter exposed the bladder, thus facilitating excision of the bladder cuff. The remaining junction of the ureter and bladder was cut off, and the bladder wall was sutured with a 3-0 Vicryl suture. A pelvic drain tube was placed, and the incision was closed.

### Follow-up after the RNU

A urinary catheter was left for approximately a week and removed after cystography. Cystoscopy and chest/abdomen/pelvis CT scans were performed approximately every 3 months for 2 years after the RNU, every 6 months from year 2 to 5, and annually thereafter.

## Results

Four patients undergoing complete laparoscopic RNU and four patients undergoing conventional RNU during the same period were included in this study. All four patients successfully underwent complete laparoscopic RNU. Clinicopathological background and surgical information are shown in Tables 1 and 2, respectively. There were no statistically significant differences in age, length of hospital stay, and follow-up duration between the two groups.

**Table 1:** A list of four patients undergoing our complete laparoscopic RNU for upper urinary tract urothelial cancer

Patient no.	Sex	Age	Tumor location	Clinical TNM^**a**^	Histology	Estimated blood loss (mL)	Pneumoperitoneum time (min)^**b**^	Pneumovesicum time (min)	Complications^**c**^	Length of hospital stay (day)	Follow-up (months)	Recurrence after RNU
1	F	77	Left, renal pelvis	T1N0M0	UC, pT1 HG	Nearly zero	171	52	None	12	25	Recurrence-free
2	M	78	Left, renal pelvis	TaN0M0	UC, pTa LG	Nearly zero	147	151	Bladder leakage (grade II)	16	15	Recurrence-free
3	M	80	Right, middle ureter	T2N0M0	UC, pT3 HG	nearly zero	202	93	Hypertension (grade I)	11	5	Recurrence-free
4	F	80	Right, upper ureter	T1N0M0	UC, pT2 HG	Nearly zero	176	57	None	11	3	Recurrence-free
Average		79					174	88		13	12	

*M* male, *F* female, *RNU* radical nephroureterectomy, *UC* urothelical carcinoma, *LG* low grade, *HG* high grade

^a^The 7th edition of the UICC-AJCC TNM staging system

^b^Pnemoperitoneum time includes nephrectomy time and specimen removal time

^c^The Clavien-Dindo classification system [9]

**Table 2:** A list of control patients undergoing conventional RNU for upper urinary tract urothelial cancer

Patient no.	Sex	Age	Tumor location	Clinical TNM^**a**^	Histology	Estimated blood loss (mL)	Pneumoperitoneum time (min)	Open surgerytime (min)	Complications^**b**^	Length of hospital stay (day)	Follow-up (months)	Recurrence after RNU
1	F	76	Right, renal pelvis	T2N0M0	UC, pT3 HG	150	98	112	None	8	24	Intravesical recurrence (5 months, Ta LG)
2	M	72	Left, middle ureter	T1N0M0	UC, pT2 HG	Nearly zero	72	117	None	12	12	Recurrence-free
3	M	84	Left, renal pelvis	T2N0M0	UC, pT3 HG	Nearly zero	78	87	Hypertension (grade I)	9	3	Recurrence-free
4	F	81	Right, renal pelvis	T1N0M0	UC, pT2 HG	185	146	85	None	11	18	Recurrence-free
Average		78					99	100		10	14	

*M* male, *F* female, *RNU* radical nephroureterectomy, *UC* urothelical carcinoma, *LG* low grade, *HG* high grade

^a^The 7th edition of the UICC-AJCC TNM staging system

^b^The Clavien-Dindo classification system [9]

Pneumoperitoneum time was longer in the complete laparoscopic RNU group compared to the conventional RNU group (174 min vs 99 min; *P* = 0.029). The pneumovesicum time of the second patient was longer than that of the other three patients because he had a history of radiotherapy for localized prostate cancer. His bladder wall did not have elasticity, and so it took longer and it was more difficult to suture the bladder wall. Cystoradiography was performed on postoperative day 6 or 7, and the urethral catheters were removed. No leakage at the transvesical port was observed. However, in the second patient, bladder leakage at the suture site of the bladder wall lasted for 2 weeks. Repeat cystoradiography demonstrated resolution of the leak, followed by removal of the urethral catheter. All patients were pathologically diagnosed with urothelial carcinoma, and none of the patients experienced recurrent disease during the follow-up (range, 3–15 months).

**Fig. 5:**
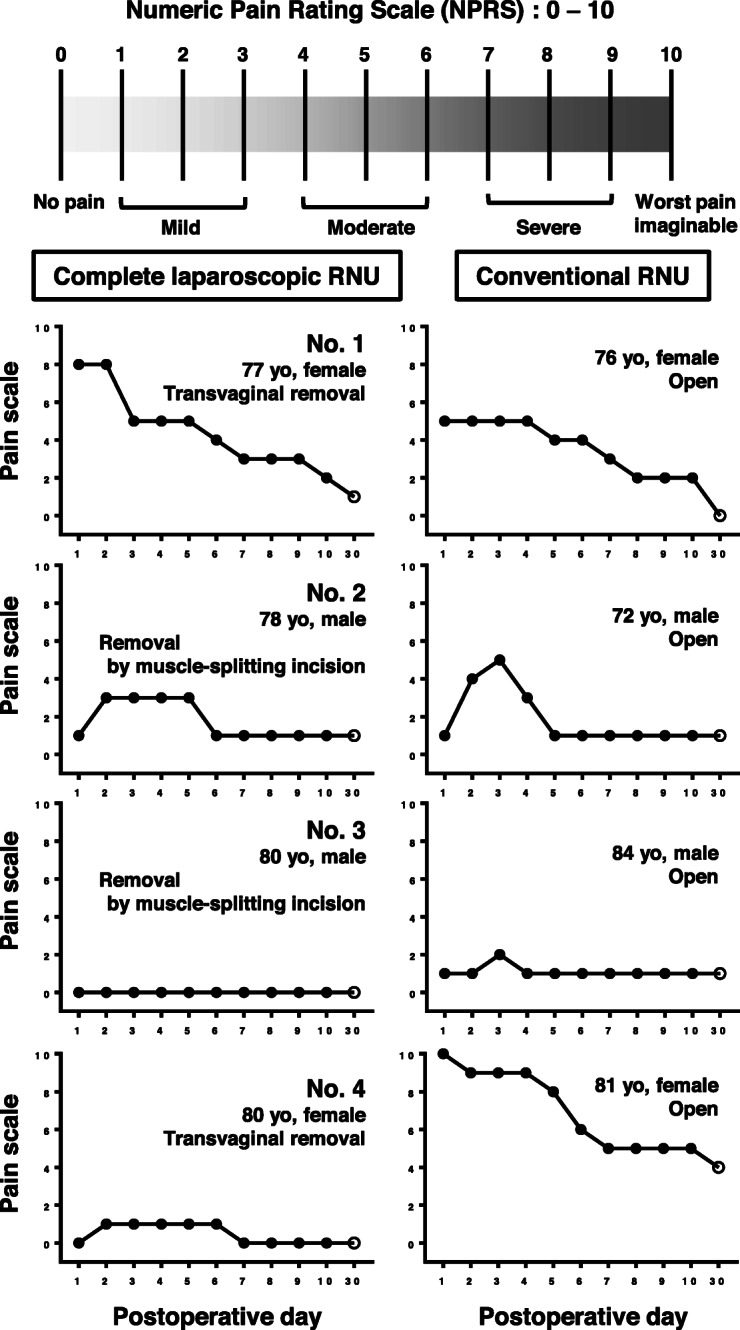
Time-course change of pain scale of complete laparoscopic nephroureterectomy and conventional nephroureterectomy. Postoperative pain was assessed using a numerical pain rating scale (NPRS) every day during the hospitalization and at the first outpatient visit day (indicated with “day 30”). The data included four patients undergoing complete laparoscopic radical nephroureterectomy (RNU), as shown in Table 1, and four patients undergoing the conventional retroperitoneal laparoscopic nephrectomy combined with an open bladder cuff by the same surgeon, during the same period, as shown in Table 2

Time-course changes in the NPRS of the complete laparoscopic RNU group (*n* = 4) and conventional RNU group (*n* = 4) are plotted in Fig. 5. Generally, mild to moderate pain lasted for 5 or 6 days after RNU. A couple of days after surgery, the NPRS of the complete laparoscopic RNU and conventional RNU groups reached peak levels at 3.0 ± 1.8 and 5.3 ± 2.8, respectively. There was no statistical difference in the degree of postoperative pain (*P* = 0.31, Mann-Whitney *U* test) in our study cohort.

## Discussion

Recent advances in laparoscopic skills and devices have enabled a safe and minimally invasive procedure by visual magnification, providing accurate suture and anastomosis. In this study, we report the initial experience of complete laparoscopic RNU with transvesical laparoscopic bladder cuff excision in patients with UTUC. Several techniques, especially in excision of the distal ureter, have been reported to date [13–16]. However, we should carefully consider the application of laparoscopic surgery in patients with UTUC, which is known as one of the most aggressive malignancies. During the follow-up, no patients experienced any recurrent disease including intravesical, extravesical, distant, and vaginal recurrence. As to the learning curve, apparent progress was not observed in terms of operation time. Longer follow-up and larger sample size are mandatory to clarify the real benefit of this surgical method.

Maximal effort should be made to ameliorate postoperative pain, shorten hospitalization stay, prevent perioperative complications, and maintain cosmesis. Based on the initial experience of our four patients, we considered our novel trial of complete laparoscopic RNU as an acceptable method (Table 1). The intravesical space is limited and can be continually obscured by effluxing urine. To counter this problem, the retroperitoneal procedure, including ligation of the renal artery and ureter, proceeds the transvesical laparoscopic procedure in our operation strategy. This can provide an additional possible advantage that occluding the ureter results in decreased risk of dissemination of tumor cells from the upper urinary tract to the intravesical cavity and paravesical space. The biggest concern with transurethral excision of the distal ureter, the so-called pluck technique, is the risk of spillage of urine containing tumor cells [17].

In a case report by Sotelo et al. [16], they performed a transvesical single-site surgery enabling full-thickness incision of the bladder around the ureteral orifice, followed by a water-tight suture of the bladder wall defect. Transvesical single-site surgery requires insertion of a special device into the bladder and subsequent suture of the bladder anterior wall. In contrast to single-site surgery, the out method does not require suture of the bladder port site because intravesical trocars are thin (only 5 mm thickness). The technique for dissection of the intramural ureter and intracorporeal suturing seems to be challenging and time-consuming, even for a laparoscopically experienced surgeon. In our second patient (Table 1), the intravesical laparoscopic procedure took no less than 2.5 h, mainly due to a history of radiotherapy for prostate cancer, which could induce ischemic conditions around the bladder trigon and decreased elasticity of the bladder wall. Eventually, he suffered from prolonged urethral catheterization. Based on this experience, a history of pelvic radiotherapy may be a potential contraindication for transvesical laparoscopic bladder cuff excision.

We applied three 5-mm trocars to the intravesical space, which may lead to a risk of port-site recurrence. No patients experienced port-site recurrence during the follow-up period in our cohort. Other options for laparoscopic excision of the distal ureter and bladder cuff are as follows: thermo-sealing system [18], cold excision of the bladder cuff with intracorporeal suturing [14, 15], modification with a bulldog clamp [19], purse-string technique [20], and robot-assisted surgery [21]. As pointed out previously, some extravesical approaches have the potential risk of failing to resect the intramural ureter. Our peumovesicoscopic bladder cuff excision ensures complete resection of the ureteral orifice, intramural ureter, and ureterovesical junction. This advantage strengthens our novel method. The transvesical laparoscopic bladder cuff technique has not yet been standardized.

We applied transvaginal specimen extraction in two female patients (cases. 1 and 4 in Table 1). We believe that the potential benefits of our method over conventional RNU could include decreased incisional pain, reduction in postoperative pain, and improved cosmesis. Since the first preclinical model of natural orifice transluminal endoscopic surgery (NOTES) nephrectomy in the urologic field [22], this approach has attracted much interest from urologists. Transvaginal NOTES has been introduced into donor nephrectomy and nephrectomy for renal infection, renal calculus, and renal malignancies [23–26]. In the literature, the multiaccess alternative of pure NOTES is often mentioned as “Hybrid NOTES,” in which standard laparoscopic nephrectomy is performed and the vagina is only utilized for specimen extraction.

This study has several limitations. Given that the decision between complete laparoscopic RNU or conventional RNU was made by physicians rather than through randomization, there is a potential selection bias. Second, the sample size was small, with only four patients in the complete laparoscopic RNU group and four patients in the conventional RNU group, and the follow-up time was short. We could not make conclusion regarding oncological outcome and benefit. However, we believe the potential of functional outcomes including decreased postoperative pain and improved cosmesis. This report can emphasize on the safety and feasibility of the new surgical method, which lead to our prospective multi-institutional trial in the near future. Third, no assessment was conducted regarding patient-reported outcomes such as health-related quality of life and sexual satisfaction questionnaire pre- and post-operatively, especially in female patients.

## Conclusion

We reviewed our experience with complete laparoscopic RNU for patients with UTUC. Although there have been many reports on the feasibility of complete laparoscopic surgery, few studies have reported on transvesical three-port bladder cuff excision. Further experience and research are mandatory to determine whether this advanced laparoscopic technique yields better outcomes and has true clinical value.

## Data Availability

All data generated or analyzed during this study are included in this published article and reference articles.

## Abbreviations

CT: Computed tomography
NOTES: Natural orifice translumenal endoscopic surgery
NPRS: Numeric pain rating scale
RNU: Radical nephroureterectomy
UTUC: Upper urinary tract urothelial cancer
